# IVUS-guided versus OCT-guided PCI among patients presenting with acute coronary syndrome

**DOI:** 10.1186/s43044-023-00377-y

**Published:** 2023-06-14

**Authors:** Mostafa Abdelmonaem, Abdelrahman Abushouk, Ahmed Reda, Sherif Arafa, Hisham Aboul- Enein, Ahmed Bendary

**Affiliations:** 1 Cardiology Department, Ain Shams University, Cairo, Egypt; 2 Department of Cardiovascular Medicine, Heart, Vascular and Thoracic Institute, Cleveland Clinic Foundation, Cleveland, OH USA; 3 Cardiology Department, Mansoura University, Mansoura, Egypt; 4 Cardiology Department, Benha University, Benha, Egypt

**Keywords:** Acute coronary syndrome, Optical coherence tomography, Intravascular ultrasound, Stent expansion

## Abstract

**Background:**

Intravascular imaging modalities such as intravascular ultrasound (IVUS) and, more recently, optical coherence tomography (OCT) improved the visualization of coronary anatomy and plaque pathology. We aimed to compare the procedural and short-term outcomes between IVUS-guided and OCT-guided percutaneous coronary interventions (PCIs) in patients with acute coronary syndrome (ACS).

**Methods:**

In the present retrospective study, we reviewed the data of 50 patients who had IVUS-guided PCI and 50 patients who had OCT-guided PCI for ACS between January 2020 and June 2021. Intravascular imaging was done before and after stenting. Both groups were compared in terms of minimal luminal area (MLA), stent dimensions, final minimal stent area (MSA) and stent expansion as well as negative angiographic outcomes. Patients were followed for six months to record major adverse cardiac events (MACE).

**Results:**

The patients’ mean age was 57 ± 13 years with male predominance (78%). The radiation time and dose were significantly higher among IVUS group. Pre-stenting MLA was significantly higher in IVUS group (2.63 mm vs. 2.22 mm in OCT, *P* = 0.013). Stent expansion was significantly higher among OCT group (97% vs. 93% in IVUS group, *P* = 0.001) with no significant difference between both groups regarding MSA [mm^2^] (8.88 ± 2.87 in IVUS vs. 8.1 ± 2.76 in OCT, *P* = 0.169). No significant difference between both groups was noted regarding contrast volume, edge dissection, tissue prolapse, and no reflow. The rates of six-month MACE were significantly higher in the IVUS group.

**Conclusions:**

OCT-guided PCI in ACS is safe and is associated with similar MSA to that of IVUS-guided PCI. Future randomized trials are needed to confirm these findings.

**Graphical Abstract:**

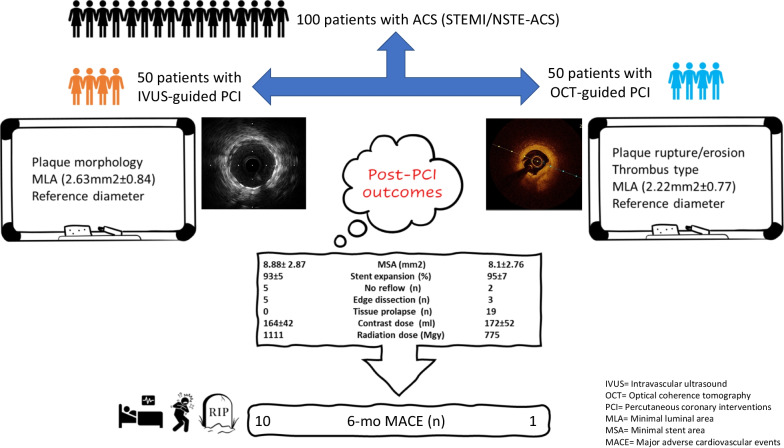

## Background

Intravascular imaging modalities such as intravascular ultrasound (IVUS) and, more recently, optical coherence tomography (OCT) were developed to improve the visualization of coronary anatomy and plaque pathology in order to optimize the outcomes of percutaneous coronary intervention (PCI) procedures [1, 2]. Pre-PCI, IVUS can accurately estimate minimal lumen area, plaque composition, calcium burden and prevention of geographic miss. Post-PCI, IVUS can precisely calculate minimal stent area (MSA), estimate stent expansion, detect tissue prolapse, malapposition and edge dissection. PCI under IVUS guidance is associated with lower incidence rates of myocardial infarction, death, and target vessel revascularization [3–6].

More recently, frequency domain OCT was introduced to overcome the limitations of time domain OCT, such as the requirement of proximal vessel occlusion, complex image acquisition and vessel under-sizing as compared to IVUS [7, 8]. Frequency domain OCT is a light-based technology with 10 times higher axial resolution as compared to IVUS with limited tissue penetration (1–2 mm). The blessing of such resolution enables interventional cardiologists to understand the concept of unstable coronary atherosclerotic plaque and introduces new definitions such as plaque rupture, plaque erosion, white thrombi, red thrombi, and thin cap fibroatheroma. Thus, OCT has a higher reproducibility as compared to IVUS [9–11].

OCT is, however, inferior to IVUS in terms of necessity of blood clearance as infra-red wavelength is much less than red blood cell wavelength, the ability of IVUS to visualize the full thickness of coronary arteries and consequently detecting remodeling process, limited accuracy in aorto-ostial lesions and excess contrast utilization as 17–70 ml of contrast is necessary to clear luminal blood [12–19].

Previous real-world studies paved the way for frequency domain OCT to be an alternative to IVUS with higher resolution and faster image acquisition [14, 20]. However, the feasibility, safety, and reproducibility of OCT in the setting of ACS are still not well-investigated. Accordingly, we aimed to compare the procedural outcomes between IVUS-guided and OCT-guided PCI in patients presenting with ACS.

## Methods

### Study design

This is a single-center retrospective study. Between January 2021 and June 2021, we reviewed the data of 100 ACS patients: 50 patients who underwent IVUS-guided PCI (group 1) and 50 patients who underwent OCT-guided PCI (group 2). The exclusion criteria were post-coronary artery bypass graft (CABG) patients, patients with end stage renal disease, poor image acquisition, vasospastic angina, and embolic coronary occlusion. Written informed consent was waived due to the retrospective design of this study and masking of all patients’ identifiers. The study was approved by the institutional review board of Saudi German hospitals.

All patients were subjected to full history taking and thorough clinical examination with emphasis on risk factors of atherosclerosis. ST-segment elevation myocardial infarction (STEMI) was defined as persistent chest pain for at least 30 min, arrival to PCI capable center within 24 h from symptom onset, ST-segment elevation > 0.1 mV in two or more contiguous leads or newly discovered left bundle-branch block. Non-STEMI was defined as prolonged chest pain with elevated cardiac biomarkers in the absence of ST-segment elevation on 12-leads surface ECG [21].

During hospital stay, pre- and post-PCI serum creatinine was withdrawn, and full echocardiographic analysis was done. Total days of hospital stay were recorded, together with immediate post-PCI adverse outcomes. Recruited patients were followed for 6 months to detect 6-month MACE (cerebrovascular events, heart failure, ischemic myocardial events and cardiac death) [22]. Scheduled follow-up for all patients was achieved through physical attendance in outpatient clinics or via phone communication spanning over 6 months from June 2021 to December 2021.

### Patient preparation

Anti-thrombotics were administered according to the latest PCI guidelines [21]. Second and third generations drug eluting stents (DES) were inserted according to the operators’ preferences. Lesion measurements were done in the worst looking view and end diastolic frames were selected, after administering 200 µg of nitroglycerin (if ABP permits).

### OCT examination

A commercially available frequency domain OCT system (Ilumien System, Light Lab Imaging, Inc., St. Jude Medical, Westford, MA, USA) and a 0.014-inch imaging wire (Image Wire, St. Jude Medical, Westford, MA, USA) were used. Motorized wire pull-back at 10 mm/s was performed during contrast injection. The PCI procedure was guided by OCT specialist mentoring lesion measurements and analysis [23].

**Fig. 1:**
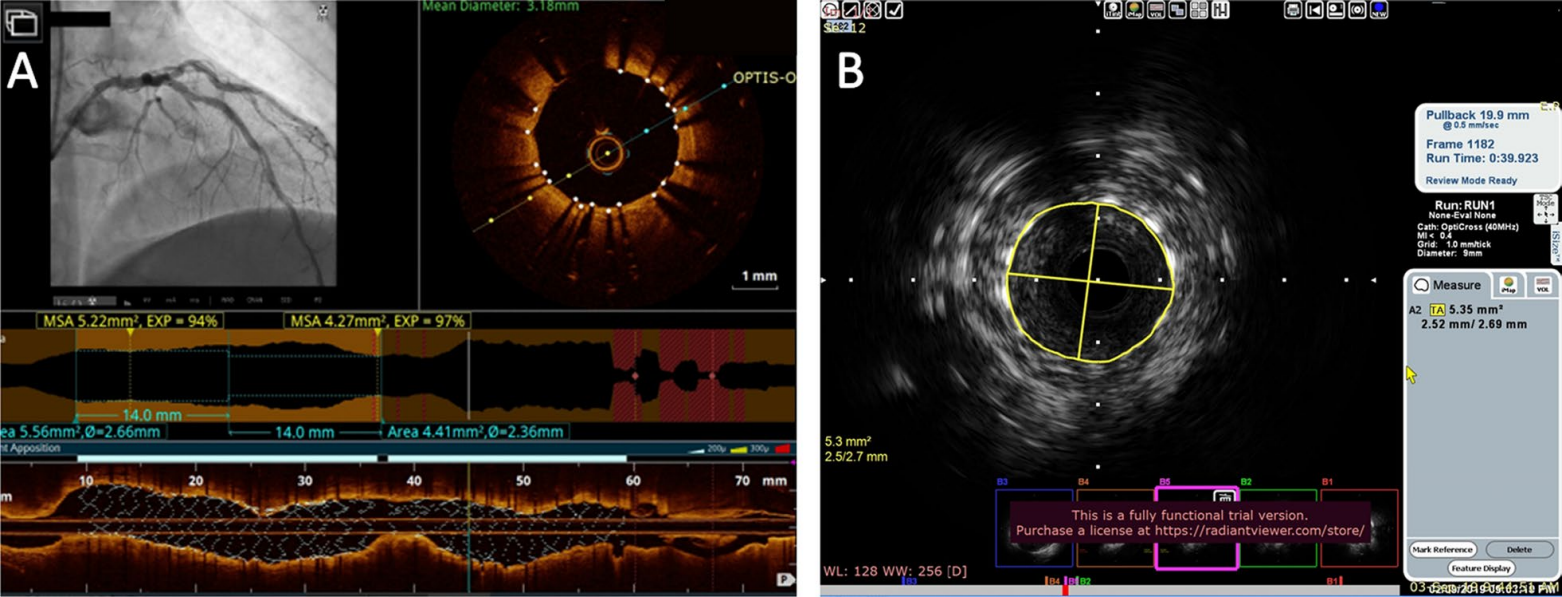
**A** OCT image in a patient with deployed stent in the LAD showing well apposed stent struts with MSA of 5.2 mm and expansion of 94% **B** IVUS image showing adequate stent apposition and expansion and MSA of 5.3 mm. *OCT* optical coherence tomography, *IVUS* intravascular ultrasound, *LAD* left anterior descending coronary, *MSA* minimal stent area

The underlying plaque morphology was identified through every frame in the culprit lesion; plaque rupture was defined as disruption of the lesion fibrous cap with plaque cavitation. Plaque erosion was defined as luminal irregularities with thrombus formation overlying an intact fibrous cap. The thrombus, if present, was categorized into red and white thrombi guided by its morphology, signal attenuation and back scattering [24, 25] (Fig. 1A).

### IVUS examination

Grayscale IVUS was performed after administration of intracoronary nitroglycerin using commercially available system, (40-MHz IVUS Catheter, Boston Scientific Corporation). The IVUS catheter was advanced distal to the lesion and then pullback 0.5 mm/sec to aorto-ostial junction. Chromaflow imaging was used if significant malapposition or edge dissection was suspected. Offline image analysis was done in specialized IVUS core lab [26] (Fig. 1B).

### Image analysis

Image analysis included lumen areas at the proximal and distal references (defined as the frames just proximal or distal to stent edges), minimum lumen areas (MLA) in the culprit segment, minimum stent area (MSA) and acute luminal gain. We did not use a definite a cut-off value for the final MSA, and it was correlated with the referenced vessel area; a final MSA ≥ 90% was considered optimal and ≥ 80% was considered acceptable. Dissection was defined as intimal disruption of the luminal surface at the stent edges, stent malapposition was defined as the presence of gap between struts and luminal surface and was considered significant if the stent-lumen distance was > 0.2 mm, and tissue prolapse was defined as tissue or thrombus protrusion between stent struts toward the lumen. TIMI (Thrombolysis in Myocardial Infarction) flow grade post-procedure was documented with special emphasis on No-reflow cases [27–31].

### Sample size calculation

The sample size was calculated using G*power software version 3.1.9.2 based on a previous study by Maoto et al. The study reported a high effect size (*d* = 0.904) of diffuse stent expansion between the IVUS and OCT groups. The total sample size calculated was 60 patients (30 per group). Alpha and power were adjusted at 0.05 and 0.9, respectively [17].

### Statistical methods

Data management and statistical analysis were done using SPSS version 25 (IBM, Armonk, New York, United States). According to normality testing, numerical data were summarized as means and standard deviations or medians and interquartile ranges (IQRs). Categorical data were summarized as numbers and percentages. Quantitative data were compared between study groups using independent *t* test or Mann–Whitney U test for normally and non-normally distributed numerical variables, respectively. Categorical data were compared using the Chi-square or Fisher’s exact test, if appropriate. Stepwise logistic regression analysis was done for the prediction of no-reflow or dissection. The odds ratio and 95% confidence interval were calculated. All statistical tests were two-sided. *P* values less than 0.05 were considered significant.

## Results

### Baseline characteristics

A hundred patients were analyzed in the present study. The mean age was higher in the IVUS group than in the OCT group (61 vs. 53 years; *p* = 0.002). The IVUS group had more females and more patients with diabetes, dyslipidemia and peripheral vascular disease than the OCT group. While the OCT group had more smokers. Both groups had similar frequencies of hypertension (*p* = 0.53) and history of cerebrovascular accidents (*p* = 0.18). The baseline creatinine levels were higher in the IVUS group than the OCT group (1.3 vs. 0.9 mg/dL, respectively; *p* < 0.001). The detailed baseline characteristics are illustrated in (Table 1).

**Table 1:** General characteristics of study population

		Total (*n* = 100)	IVUS (*n* = 50)	OCT (*n* = 50)	*P* value
Age (years)	Mean ± SD	57 ± 13	61 ± 14	53 ± 12	**0.002**
Gender	Males *n* (%)	78 (78)	34 (68)	44 (88)	**0.016**
	Females *n* (%)	22 (22)	16 (32)	6 (12)	
Smoking	*n* (%)	67 (67)	25 (50)	42 (84)	** < 0.001**
Hypertension	*n* (%)	65 (65)	34 (68)	31 (62)	0.529
Diabetes mellitus	*n* (%)	46 (46)	29 (58)	17 (34)	**0.016**
Dyslipidemia	*n* (%)	59 (59)	35 (70)	24 (48)	**0.025**
PVD	*n* (%)	6 (6)	6 (12)	0 (0)	**0.027**
CVA	*n* (%)	10 (10)	7 (14)	3 (6)	0.182
Baseline creatinine (mg/dl)	Mean ± SD	1.1 ± 0.4	1.3 ± 0.5	0.9 ± 0.1	** < 0.001**
Presentation	Anterior STEMI *n* (%)	38 (38)	14 (28)	24 (48)	**0.01**
	Inferior STEMI *n* (%)	13 (13)	4 (8)	9 (18)	
	Non-STEMI *n* (%)	49 (49)	32 (64)	17 (34)	
Killip	I *n* (%)	42 (82)	9 (50)	33 (100)	** < 0.001**
	II *n* (%)	4 (7.8)	4 (22.2)	0 (0)	
	III *n* (%)	0 (0)	0 (0)	0 (0)	
	IV *n* (%)	5 (9.8)	5 (27.8)	0 (0)	
Time to 1st medical contact (hrs)	Median (IQR)	10 (6–17.5)	12 (8–19)	6.5 (5–12)	**0.001**

Bold value denotes statistically signicifant differences

Independent *t* test was used for age and creatinine. Mann–Whitney *U* test was used for time to 1st medical contact. Chi-square or Fisher’s exact was used for categorical data

*PVD* peripheral vascular disease, *CVA* cerebrovascular accident

### Clinical findings

The rate of non-STEMI was higher in the IVUS group compared to the OCT group (64 vs. 34%), while the rate of STEMI (anterior and inferior) was higher in the OCT group (66 vs. 36%; *p* = 0.01). The most frequent Killip class was I (82.0%), followed by IV (9.8) and II (7.8%). Killip class showed a significant difference between both groups (*p* < 0.001); Killip I was more frequent in the OCT group, while Killip II and IV were more frequent in the IVUS group (Table 1).

### Procedural characteristics

The median radiation dose (1111 vs. 775 Mgy; *p* = 0.008) and mean radiation time (13.3 vs. 10.7 min; *p* = 0.002) were significantly higher in the IVUS group than the OCT group. Regarding the mean expansion, it was 95% in the studied patients, and it was significantly higher in the OCT group than the IVUS group (97 vs. 93%; *p* = 0.001); *p* was 0.001. Similarly, the mean MLA was significantly higher in the IVUS group than in the OCT group (2.63 vs. 2.22; *p* = 0.013).

No significant differences were noted between both groups in terms of the average stent diameter (*p* = 0.111), average stent length (*p* = 0.306), contrast dose (*p* = 0.432), minimal stent area (MSA) (*p* = 0.169), acute MLA gain (*p* = 0.415), acute luminal gain (ALG)% related to reference vessel area (*p* = 0.117), as well as the rates of no-reflow (*p* = 0.436) and dissection (*p* = 0.715) (Table 2).

**Table 2:** Procedural characteristics of the study groups

		Total (*n* = 100)	IVUS (*n* = 50)	OCT (*n* = 50)	*P* value
Average stent diameter (mm)	Mean ± SD	3.3 ± 0.8	3.4 ± 1	3.2 ± 0.6	0.111
Average stent length (mm)	Mean ± SD	27.2 ± 9.2	26.3 ± 8.4	28.2 ± 9.9	0.306
Contrast (ml)	Mean ± SD	168 ± 47	164 ± 42	172 ± 52	0.432
Radiation dose (Mgy)	Median (range)	942 (651–1403)	1111 (737–1632)	775 (563–1154)	**0.008**
Rad time (min)	Mean ± SD	12 ± 4.3	13.3 ± 4.3	10.7 ± 3.9	**0.002**
No reflow	*n* (%)	7 (7.0)	5 (10)	2 (4.0)	0.436
Prolapse	*n* (%)	–	–	19 (38)	–
Dissection	*n* (%)	8 (8.0)	5 (10)	3 (6)	0.715
Expansion (%)	Mean ± SD	95 ± 6	93 ± 5	97 ± 7	**0.001**
MLA (mm^2^)	Mean ± SD	2.42 ± 0.83	2.63 ± 0.84	2.22 ± 0.77	**0.013**
MSA (mm^2^)	Mean ± SD	8.49 ± 2.83	8.88 ± 2.87	8.1 ± 2.76	0.169
Acute MLA gain (mm^2^)	Mean ± SD	6.05 ± 2.48	6.26 ± 2.41	5.85 ± 2.56	0.415
ALG % related to ref versus area	Mean ± SD	63.3 ± 12.6	61.3 ± 10	65.2 ± 14.6	0.117
Plaque character	Fibrotic *n* (%)	–	12 (24)	–	–
	Calcification *n* (%)	–	12 (24)	–	
	Necrotic *n* (%)	–	26 (52)	–	
Acute plaque change type	Ruptured *n* (%)	–	–	32 (64)	–
	Erosion *n* (%)	–	–	16 (32)	
	SCAD *n* (%)	–	–	2 (4)	
Type of thrombus	Red *n* (%)	–	–	22 (44.9)	–
	White *n* (%)	–	–	27 (55.1)	

Bold value denotes statistically signicifant differences

Independent *t* test or Mann–Whitney *U* test was used for numerical data. Fisher’s exact was used for no-reflow and dissection

*MLA* minimum luminal area, *MSA* minimum stent area, *ALG* acute luminal gain

### Outcome

The median hospital stay was significantly longer in the IVUS group than the OCT group (3 vs. 2 days; *p* < 0.001). Moreover, the median post-procedure creatinine level was significantly higher in the IVUS group than in the OCT group (1.2 vs. 1 mg/dl; *p* < 0.001). At six months, 10 patients (20%) experienced MACE in the IVUS group, compared to only one (2%) in the OCT group (*p* = 0.004) (Table 3).

**Table 3:** Outcome of the study groups

		Total (*n* = 100)	IVUS (*n* = 50)	OCT (*n* = 50)	*P* value
Hospital stay (days)	Median (IQR)	3 (2–3)	3 (2–4)	2 (2–3)	< 0.001
Creatinine post (mg/dl)	Median (IQR)	1.1 (1–1.30)	1.2 (1.1–1.58)	1 (1–1.1)	< 0.001
MACE	*n* (%)	11 (11)	10 (20)	1 (2)	0.004

Mann–Whitney U test was used for numerical data. Fisher’s exact was used for MACE

*MACE* Major adverse cardiovascular event

### Prediction of no-reflow or dissection

Stepwise logistic regression analysis was done for the prediction of no-reflow or dissection. The following variables were included: age, gender, smoking, hypertension, diabetes mellitus, dyslipidemia, peripheral vascular disease (PVD), cerebrovascular accident (CVA), modality used, presentation, stent length, stent diameter, expansion, and radiation dose. The only significant predictor that remained in the model was stent diameter (OR 2.320, 95% CI 1.166–4.616, *p* = 0.016). The use of intravascular imaging either IVUS or OCT was not an independent predictor of occurrence of no-reflow phenomenon among the studied cohort of patients.

## Discussion

The current retrospective study comparing OCT-guided to IVUS-guided PCI in patients presenting with ACS showed no statistically significant difference between both modalities in terms of post-PCI MSA. OCT-guided PCI was also associated with a significantly higher percent stent expansion compared to IVUS-guided PCI, with lower rates of no-reflow and dissections in OCT-guided PCI group. The higher resolution of OCT compared to IVUS enables for better detection of post-procedural tissue prolapses and thrombus [32]. This was evident in the current report.

Because post-PCI MSA is the most important independent predictor for long-term freedom of early and late post-procedural MACE [33], obtaining similar post-PCI MSA with OCT-guided compared to IVUS-guided techniques as we showed is clinically relevant. This is reassuring, in that the higher image resolution advantage of OCT is not negated by a lower post-PCI MSA. Adding to the robustness of the post-PCI MSA finding, it should be noted that the pre-PCI MLA of the lesions was significantly lower in the OCT-guided group.

Recently, the ILUMIEN III: OPTIMIZE PCI randomized study concluded that OCT guided PCI (using a specific reference segment external elastic lamina-based stent optimization strategy) was safe and resulted in similar MSA to that of IVUS-guided PCI. However, it included a relatively heterogenous group of patients, i.e., both elective PVI and PCI in the setting of ACS [34]. The current study, albeit non-randomized, focused exclusively on patients presenting acutely with ACS.

Another study compared the ability of OCT-guided PCI to angiographic guidance alone in improving post-procedural fractional flow reserve (FFR) in non-STEMI patients. It showed that OCT-guided PCI resulted in a significantly higher post-procedural FFR [16]. Importantly, post-PCI MSA in this study set out to be the best predictor of satisfactory post-PCI FFR (more than 0.90) with receiver operator characteristic analysis.

We also explored the difference between both intravascular imaging modalities on six months clinical outcomes (MACEs). Although the study was not powered for such an endpoint, the incidence of MACEs at 6 months was significantly higher in IVUS arm, which is not in line with a somewhat more powered multicenter randomized study (the OPINION trial) that showed similar rates of target vessel failure at 12 months between IVUS- and OCT-guided PCI strategies, but in the OPINION TRIAL, the main focus was target vessel failure and not composite MACE outcome [18].

Our finding, albeit hypothesis-generating, that OCT strategy was associated with significantly less radiation dose and a shorter radiation time is noteworthy here. In the recently published iSIGHT trial, radiation time was numerically longer in OCT group with no statistical significance [35].

Adding to the validity of the current study’s findings, and in a stepwise multivariate logistic regression model, using either IVUS or OCT for guidance of PCI among our patient’s population turned out not to be a significant independent predictor for the occurrence of no-reflow or dissection (both are important surrogate endpoints for future MACEs) [36].

The current study is not without limitations. First, this was a retrospective comparison of the two imaging strategies; therefore, selection bias cannot be excluded. Any differences in clinical MACEs at 6 months between study groups could be due to these selection bias which may have led to imbalances in the baseline characteristics between groups. Second, the lack of an angiography-guided PCI arm is a concern. Third, the study was not powered to detect differences in clinical outcomes, and thus, any differences in this domain remain hypothesis generating. Larger randomized studies with longer follow-up periods are needed to answer this clinical question.

Despite these limitations, we believe our data add to the body of evidence supporting the use of OCT-guided PCI to optimize procedural outcomes in different PCI scenarios. That’s because, as we showed, OCT seems to provide higher resolution that translates into better detection of post-procedural tissue prolapses and thrombi, and this is accomplished without sacrificing post-PCI MSA or clinical outcomes.

## Conclusions

OCT-guided PCI in patients with ACS is safe and associated with no statistically significant difference in post-PCI MSA compared to that of IVUS-guided PCI. Further prospective randomized studies are needed to corroborate these findings.

## Data Availability

All data are available upon reasonable request from the corresponding author(s).

## Abbreviations

ACS: Acute coronary syndrome
CABG: Coronary artery bypass grafting
DES: Drug-eluting stents
IQRs: Inter-quartile ranges
IVUS: Intravascular ultrasound
MACEs: Major adverse cardiovascular events
MLA: Minimal luminal area
MSA: Minimal stent area
OCT: Optical coherence tomography
PCI: Percutaneous coronary intervention
STEMI: ST-elevation myocardial infarction
